# The Longitudinal Transcriptomic Response of the Substantia Nigra to Intrastriatal 6-Hydroxydopamine Reveals Significant Upregulation of Regeneration-Associated Genes

**DOI:** 10.1371/journal.pone.0127768

**Published:** 2015-05-20

**Authors:** Nicholas M. Kanaan, Timothy J. Collier, Allyson Cole-Strauss, Tessa Grabinski, Zachary R. Mattingly, Mary E. Winn, Kathy Steece-Collier, Caryl E. Sortwell, Fredric P. Manfredsson, Jack W. Lipton

**Affiliations:** 1 Department of Translational Science & Molecular Medicine, College of Human Medicine, Michigan State University, Grand Rapids, MI, United States of America; 2 Morris. K. Udall Center of Excellence in Parkinson’s Disease Research, Michigan State University, Grand Rapids, MI, United States of America; 3 Hauenstein Neuroscience Center, Mercy Health Saint Mary’s, Grand Rapids, Michigan, United States of America; 4 Bioinformatics & Biostatistics Core, Van Andel Research Institute, Grand Rapids, MI, United States of America; St. Jude Children's Research Hospital, UNITED STATES

## Abstract

We hypothesized that the study of gene expression at 1, 2, 4, 6 and 16 weeks in the substantia nigra (SN) after intrastriatal 6-OHDA in the Sprague-Dawley rat (*rattus norvegicus*) would identify cellular responses during the degenerative process that could be axoprotective. Specifically, we hypothesized that genes expressed within the SN that followed a profile of being highly upregulated early after the lesion (during active axonal degeneration) and then progressively declined to baseline over 16 weeks as DA neurons died are indicative of potential protective responses to the striatal 6-OHDA insult. Utilizing a κ-means cluster analysis strategy, we demonstrated that one such cluster followed this hypothesized expression pattern over time, and that this cluster contained several interrelated transcripts that are classified as regeneration-associated genes (RAGs) including Atf3, Sprr1a, Ecel1, Gadd45a, Gpnmb, Sox11, Mmp19, Srgap1, Rab15,Lifr, Trib3, Tgfb1, and Sema3c. All exemplar transcripts tested from this cluster (Sprr1a, Ecel1, Gadd45a, Atf3 and Sox11) were validated by qPCR and a smaller subset (Sprr1a, Gadd45a and Sox11) were shown to be exclusively localized to SN DA neurons using a dual label approach with RNAScope in situ hybridization and immunohistochemistry. Upregulation of RAGs is typically associated with the response to axonal injury in the peripheral nerves and was not previously reported as part of the axodegenerative process for DA neurons of the SN. Interestingly, as part of this cluster, other transcripts were identified based on their expression pattern but without a RAG provenance in the literature. These "RAG-like" transcripts need further characterization to determine if they possess similar functions to or interact with known RAG transcripts. Ultimately, it is hoped that some of the newly identified axodegeneration-reactive transcripts could be exploited as axoprotective therapies in PD and other neurodegenerative diseases.

## Introduction

The 6-hydroxydopamine (6-OHDA) model of Parkinson’s disease (PD) has been extensively characterized and employed to study the consequences of dopamine neuron loss and to screen therapeutics [1–9]. While there is continued debate as to the etiology of PD, it is clear that 6-OHDA rodent models remain relevant preclinical tools for examining the behavioral and biologic consequences of nigrostriatal dopamine (DA) neuron degeneration during disease progression. In this regard, our lesioning paradigm using intrastriatal administration of 6-OHDA [6–8,10] causes retrograde axonal degeneration that results in a rapid and near complete denervation of the striatum (STR) with a slower, more progressive, 76–80% loss of substantia nigra (SN) DA neurons by approximately six weeks [6,8]. Notably, a recent postmortem evaluation of nigrostriatal pathology in people who died in varying stages of PD found that near complete striatal DA axon denervation occurs within the first four years after clinical diagnosis, followed later by progressive DA neuron loss [11]. The key finding of this study was that striatal DA denervation is an early and fairly rapid process in PD. Hence, the intrastriatal 6-OHDA lesion model replicates the basic ordinal progression of the PD degenerative process, albeit in an accelerated timeframe.

Using this model, we hypothesized that the study of progressive gene expression changes over time in the SN after 6-OHDA insult would identify protective (albeit insufficient) cellular responses that are engaged during the neurodegenerative process. Subsequent studies could then assess whether artificial induction or silencing of the observed gene expression changes could be exploited to protect against DA neuronal or axonal damage. In this way, we are not attempting to identify etiologic factors of PD, but rather endeavor to exploit the natural response of DA neurons to degenerative cues in order to identify new manipulatable targets to repair, stabilize, or interfere with the neurodegenerative process of SN DA neurons.

The current study examined the progressive gene expression changes over 16 weeks in the SN after an intrastriatal 6-OHDA lesion. We hypothesized that patterns of gene expression within the SN characterized by early, highly upregulated expression would be indicative of potential protective responses to a striatal 6-OHDA insult. We additionally hypothesized that the use of k-means cluster analysis to group transcripts based upon expression patterns would aid in the identification of previously unknown neuroprotective candidates. Our results demonstrated that intrastriatal 6-OHDA treatment upregulated several interrelated transcripts classified as members of the regeneration-associated gene (RAGs) family that segregated into one cluster. Upregulation of RAGs is historically associated with the response to axonal injury in the peripheral nerves [12–15] and was not previously associated with axonal degeneration of SN DA neurons. In addition, other transcripts displaying a similar regulation pattern were identified and should be examined for similar function or interaction with the RAG family. These clustered RAG and RAG-like transcripts should be examined for their role in neurodegenerative and neuroprotective processes in SN DA neurons.

## Materials and Methods

### Subjects

Male, Sprague-Dawley rats (*rattus norvegicus*, Harlan, Indianapolis, IN) weighing between 200–225 g (approximately 60–75 days old) were given access to food and water *ad libitum*, and housed in an AAALAC accredited animal facility on a standard light-dark cycle. Housing conditions and animal procedures were performed according to the Association for Assessment and Accreditation of Laboratory Animal Care International guidelines and with the approval of the Michigan State University Institutional Animal Care and Use Committee. This study was carried out in strict accordance with the recommendations in the Guide for the Care and Use of Laboratory Animals of the National Institutes of Health. All surgery was performed under sodium pentobarbital anesthesia, and all efforts were made to minimize suffering. Animals were divided into the following experimental conditions: No surgery control (WK 0), unilateral intrastriatal 6-hydroxydopamine lesion (6-OHDA) or sham surgical controls (SHAM). SHAM and 6-OHDA subjects were sacrificed at 5 different times post-lesion: 1 (WK1), 2 (WK2), 4 (WK4), 6 (WK6) or 16 (WK16) weeks post-surgery. Three subjects from each of the 11 groups (5 time points x 2 treatments + the 1 NAÏVE group) who met all inclusion criteria (see below) were subjected to microarray analyses. The quantitative real-time PCR (qPCR) validation was conducted in the same cohort that was subjected to microarray analyses. Subjects utilized for immunohistochemistry and *in situ* hybridization (ISH) were sacrificed at 1 week post-lesion.

### Unilateral intrastriatal 6-OHDA lesion

Rats were anesthetized with equithesin (0.3ml/100 g of weight, i.p; chloral hydrate 42.5 mg/ml + sodium pentobarbital 9.72 mg/ml) and placed into a stereotaxic frame. Each rat received two injections of 6-OHDA HBr [Sigma-Aldrich, St. Louis, MO; 5 μg/μl, calculated as free base in 0.2% ascorbic acid and a 0.9% saline solution] at 2 μl per site. The two injection coordinates were (i) AP +1.6 mm, ML +2.4 mm, DV −4.2mm, (ii) MP +0.2 mm, ML +2.6 mm, DV −7.0mm. For all rats, the needle was zeroed at the skull directly above the injection site in order to target the DV coordinate. For each injection, the needle was lowered slowly to the injection site and 1 minute elapsed before injection commenced, 6-OHDA was injected at 0.5 μl/min and at the end of the injection the needle remained in place for an additional 4 minutes before retraction.

### Tissue collection

At the appropriate post-surgical time point, rats were anesthetized with pentobarbital (50mg/kg intraperitoneally), and decapitated. Brains were removed rapidly and submerged for 30 s in a 250ml beaker of isopentane chilled in powdered dry ice. The brains were then wrapped in foil and stored at -80°C until dissected. Frozen brains were slabbed on an inverted petri dish over a bed of crushed ice. Slabs containing the striatum were dissected using scalpels. A small portion (~ 2mm^3^) of the striatum from each side of the brain was separately reserved for confirmation of lesion status using HPLC (see below). Tissue from the substantia nigra (SN) was placed in 1 ml of trizol, (Life Technologies, Carlsbad, CA), homogenized by hand with a disposable plastic pestle, frozen on dry ice and stored at -80°C in preparation for RNA isolation

### High performance liquid chromatography (HPLC)

Striatal DA levels (i.e. 6-OHDA lesion status) were quantified by HPLC as described previously[16–18]. Briefly, samples were sonicated into 150 μl (SN) or 250 μl (STR) of a 0.4 N perchlorate, 1.34 mM EDTA and 0.53 mM sodium metabisulfite solution. A 20 μl aliquot of the homogenate was reserved for protein determination and the remaining homogenate was centrifuged at 10,500 rpm for 10 minutes at 4°C. The supernatant was stored in a separate tube at-80°C. Sample separation was performed on a 250x4.6mm Microsorb MV C18 100–5 column (Agilent, Santa Clara, CA). DA levels were detected and quantitated using a 12-channel CoulArray 5200 coulometric array detector (ESA, Chelmsford, MA). The mobile phase consisted of 100mM Citric Acid, 75mM Na_2_HPO_4_Na, 80°M 1-heptanesulfonate monohydrate, sodium salt, 5% MeOH, pH 4.25. Samples values were interpolated against a 6 point standard curve. The final values were standardized based on protein content (BCA Protein Assay Kit, Pierce Inc., Rockford, IL). Striatal DA depletion of >95% in the lesioned hemisphere as compared to the unlesioned hemisphere was used as a criterion for inclusion in the study.

### RNA isolation and quality evaluation

RNA extraction was performed using the RNA Clean and Concentrator kit (Zymo Research, Irvine, CA) and eluted into 15μl H_2_O. RNA quality was assessed using the RNA Nano 6000 Assay on an Agilent Bioanalyzer, (Santa Clara, CA). RNA quality was measured using the 10-point scale associated with the RNA Integrity Number (RIN). Only samples with RIN values ≥ 7 qualified for inclusion in microarray analyses. The mean and standard deviation of RIN values for all of the samples was 8.7 ± 0.71 (n = 33).

### Microarray sample processing and hybridization

Isolated RNA from tissue samples (n = 33) were processed for microarray hybridization on the Rat Gene 1.0 ST Array at the Gene Expression Microarray Core of Cincinnati Children’s Hospital Medical Center, Cincinnati, OH. 50-120ng of total RNA was converted to biotin–labeled sense-strand cDNA for hybridization using the Ambion WT Expression Kit (Life Technologies, Carlsbad, CA) combined with the GeneChip WT Terminal Labeling Kit (Affymetrix, Santa Clara, CA). Chips were incubated at 45°C for 17 hours in the GeneChip Hybridization Oven 640, washed and stained in the Fluidics Station 450 (Affymetrix, Santa Clara, CA), and scanned using an Affymetrix Gene Chip Scanner 3000 7G (Affymetrix, Santa Clara, CA).

### Microarray image analysis and quality control

Only array images meeting all of the quality control measures defined by the Affymetrix Expression Control Program were included in this study. Specific quality control metrics included signal histogram, relative log expression signal, Pearson’s correlation, PM mean (average signal intensity of probes), and positive and negative area under the curve (AUC). Also measured were the expression values of spiked-in poly-A RNA controls, and values of spiked-in hybridization controls. Raw data are available on the NCBI Gene Expression Omnibus Repository (http://www.ncbi.nlm.nih.gov/geo [#GSE58710])

### Analysis of microarray data

Data processing and analysis were performed using R v3.0.2 (http://www.R-project.org) and Bioconductor [19]. Affymetrix-formatted CEL files were imported into R, assessed for quality, and Robust-Multi Array (RMA) normalized at the transcript level using the oligo package (v1.26.0). Array quality was evaluated via visual assessment of the array images, intensity distributions, multi-array plots, and principal component analysis. Differential expression between 6-OHDA and sham rats was assessed at each time point using limma (v3.18.6). The resulting moderated t-statistics with an associated Benjamini-Hochberg false discovery rate (FDR) adjusted p-value less than 0.05 were used to identify expression patterns of interest using ConsensusClusterPlus (v1.16.0) [20]. Consensus clustering is a quantitative, unsupervised method used to assign objects into groups (i.e. clusters). Clustering is performed using the intrinsic relationships within the data and without external information. Repeated clustering using subsets of the data provides information regarding confidence in the number of groups and the genes within each group. The moderated t-statistic is the ratio of the log2-fold change to its standard error. T-statistics were utilized due to improved stability of consensus clustering as compared to log2-fold change. Cluster number (k) was chosen for cluster stability and a maximum mean cluster consensus score after an initial drop.

### Validation of microarray gene expression changes by qPCR

To validate expression differences observed from microarray analysis, qPCR was performed to quantify levels of *Sprr1a*, *Ecel1*, *Gadd45*, *Atf3*, and *Sox11*. The mRNA levels in frozen SN micropunches were derived from the same subjects at the WK2 post-surgical time point. Total RNA was converted to cDNA using Superscript VILO Mastermix (Invitrogen/Life Technologies, Carlsbad, CA). PCR reactions were run in 30 μl using target specific, FAM labeled Taqman hydrolysis probes (Applied Biosystems/Life Technologies, Carlsbad, CA) multiplexed with a VIC labeled primer-limited probe set to the *Gapdh* reference gene, which was confirmed to not vary with respect to the different treatment conditions (data not shown). Normalized gene expression was determined by differences in the cycle thresholds (Ct) between genes of interest and *Gapdh* (∆Ct) on a Viia7 qPCR System (Life Technologies). Differences between these normalized values across treatment groups (∆∆Ct) were then linearized to determine fold change differences (2^-∆∆Ct^) and compared to fold changes observed from samples subjected to microarray hybridization and quantitation.

### Pathway analysis of Cluster-1

The 94 probe sets representing 88 genes from Cluster-1 and their corresponding fold changes from WK1 (see S1 Table) were imported into MetaCore version 6.19 build 65960 (Thomson Reuters, New York, NY) for pathway and network analysis. Pathway analysis was performed using the Pathway Maps One-Click Analysis. MetaCore Pathway Maps are rigorously curated pathways supported by experimental evidence reported in the literature. An algorithm based on the hypergeometric distribution is used to calculate enrichment p-values. Pathway Maps with a FDR adjusted p-value less than 0.05 were considered significant. Network analysis was performed using the *“shortest paths”* algorithm with a maximum number of steps in the path set to 1 without the use of canonical pathways. Nodes and edges were exported from MetaCore and imported into Cytoscape v3.1.1 for final visualization.

### Tyrosine hydroxylase (TH) immunostaining and in situ hybridization (ISH) of Cluster-1 transcripts to determine colocalization

Animals were sacrificed 1 week after 6-OHDA (the peak of transcript expression in Cluster -1 and prior to significant loss of SN DA neurons). We utilized a combination of immunohistochemical (IHC) staining for TH and ISH for determining whether the highest fold-change Cluster-1 transcript, *Sprr1a*, and two transcripts known to influence *Sprr1a* expression *Sox11* and *Gadd45a* were localized to DA neurons in the SN. Briefly, the brains were cut into 20μm thick free-floating sections and sections were first processed for RNAscope ISH for *Sprr1a*, *Sox11* or *Gadd45a*. RNAscope is a commercially available branched or “tree” hybridization technology provided as a kit by Advanced Cell Diagnostics. Custom RNAscope target probes were generated against *Sprr1a* (407411, NM_021864.1, probe region 3–801), *Sox11* (414971, NM_053349.1, probe region 304–1955) and Gadd45a (414961, NM_024127.2, probe region 5–995). The ISH signal was detected using 3, 3’-diaminobenzidine to produce brown punctate staining. Following RNAscope ISH labeling, the sections were processed for TH (a DA neurons marker, Millipore, MAB318, 1:50,000) IHC using Vector slate-grey (SG) to produce blue-grey labeling of DA neurons (for a detailed protocol see [21]). DA neurons were identified both by their expression of TH and their morphology within the SN pars compacta (SNpc) as TH neurons can lose their phenotype when stressed or undergoing degeneration.

## Results

### Lesion Status Confirmation

Lesion status in the STR was quantified by the proportion of DA (ng/mg protein) from the ipsilateral (operated) to the contralateral (non-operated) side and converted to a percentage of the contralateral side. The 6-OHDA subjects that met exclusion criteria had mean DA levels of 0.8% ± 2.1% in the operated striatum (0.9 ng/mg protein) as compared to the intact side (119.1 ng/mg protein). Sham subjects had mean DA levels of 101.8% ±3.2% in the operated striatum (126.3 ng/mg protein) as compared to the non-operated striatum (124.1 ng/mg protein).

### Consequences of striatal 6-OHDA injections on transcriptomic changes in the SN over 16 weeks

#### Microarray analysis

**Fig 1:**
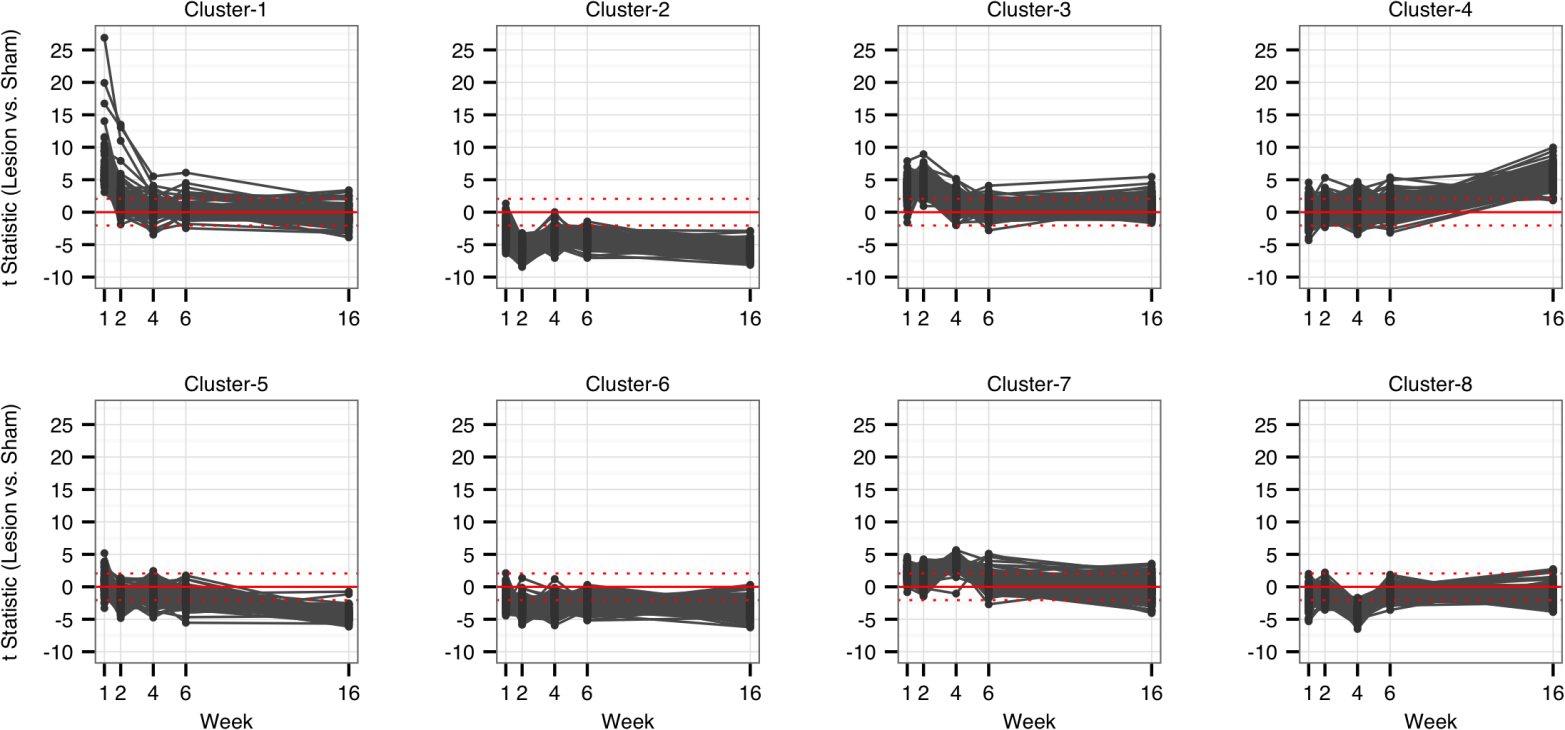
K-means cluster analysis identifies 88 genes acutely upregulated in response to lesioning. Eight clusters were identified through consensus clustering analysis. We hypothesized that a cluster of genes would be highly upregulated due to 6-OHDA administration that would fall-off over time due to ongoing DA neuron degeneration. Cluster-1 followed this hypothesized pattern.

**Fig 2:**
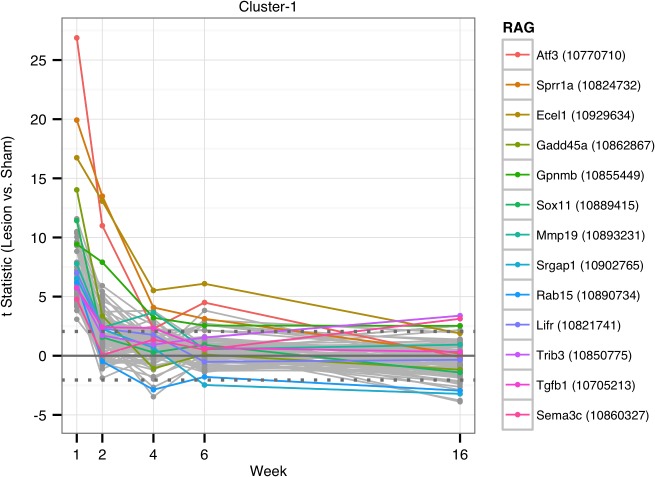
Thirteen known regeneration-associated genes are part of the 88 genes of Cluster-1. The expression pattern of the non-RAG genes follows a “RAG-like” expression pattern suggesting that they may too be part of the RAG family.

Using k-means cluster analysis we identified eight clusters of genes in the SN that covaried over time as a result of intrastriatal 6-OHDA lesion (Fig 1). For data from all clusters see S2 Table. Transcripts in Cluster-1 exhibited a robust increase one week following the 6-OHDA lesion, progressively declined at 2, 4 and 6 weeks post-lesion, and returned to basal or near basal levels by 16 weeks. The top 20 transcript changes for Cluster-1 are depicted in Fig 2 and enumerated in Table 1; significantly enriched pathways for the Cluster-1 are listed in Table 2. Interestingly, 13 transcripts within Cluster-1 fit the definition of “regeneration-associated genes” or RAGs, which identify a functional class of genes that are up-regulated in neuronal perikarya following axotomy or axonal damage *[12–15,22–35]*. These genes include *Atf3*, *Sprr1a*, *Ecel1*, *Gadd45a*, *Gpnmb*, *Sox11*, *Mmp19*, *Srgap1*, *Rab15*,*Lifr*, *Trib3*, *Tgfb1*, and *Sema3c*. The changes in expression of these RAGs in comparison to other members of the cluster are shown in Fig 2. Since the remainder of the transcripts in Cluster-1 demonstrated a similar differential expression profile, henceforth we will refer to them as “RAG-like” transcripts.

**Table 1:** Top twenty differentially expressed genes from Cluster-1.

**Transcript ID**	**Gene Symbol**	**Entrez ID**	**Fold Change**	**FDR Adj. p-value**
**10770710**	***Atf3***	25389	6.93	2.28x10^-16^
**10824732**	***Sprr1a***	499660	19.16	2.26x10^-13^
**10929634**	***Ecel1***	60417	12.97	1.11x10^-11^
**10862867**	***Gadd45a***	25112	3.31	5.79x10^-10^
**10739268**	Apoh	287774	4.35	3.75x10^-08^
**10889415**	***Sox11***	84046	2.19	4.26x10^-08^
**10937160**	Foxp3	317382	2.68	2.31x10^-07^
**10922492**	Arid5a	316327	1.87	2.73x10^-07^
**10883159**	Ucn	29151	2.90	4.73x10^-07^
**10740869**	Tnfrsf12a	302965	2.10	9.80x10^-07^
**10722097**	Tph1	24848	2.32	1.30x10^-06^
**10855449**	***Gpnmb***	113955	2.39	1.30x10^-06^
**10836504**	Xirp2	311098	1.77	1.31x10^-06^
**10726408**	Mki67	291234	2.06	1.33x10^-06^
**10839655**	Bcl2l11	64547	1.84	1.39x10^-06^
**10742145**	Ccng1	25405	1.63	3.77x10^-06^
**10711017**	Itgal	308995	1.67	2.93x10^-05^
**10717803**	Ipcef1/Oprm1	361474/25601	1.85	2.93x10^-05^
**10931717**	C3	24232	2.07	2.93x10^-05^
**10893231**	***Mmp19***	304608	1.92	3.45x10^-05^

Seven of the top twenty differentially expressed genes from the substantia nigra ipsilateral to intrastriatal injections (6-OHDA or vehicle) from Cluster-1 at 1 week post-surgery are part of the regeneration-associated (RAG) gene family (identified here in ***bold italics***).

**Table 2:** Significantly enriched pathways in Cluster-1.

**Pathway**	**p-value**	**FDR**	**Ratio**
**Cell cycle: Transition and termination of DNA replication**	3.074x10^-4^	1.687x10^-2^	3/28
**Development: Transcription factors in segregation of hepatocytic lineage**	3.784x10^-4^	1.687x10^-2^	3/30
**Cell cycle: Role of APC in cell cycle regulation**	4.593x10^-4^	1.687x10^-2^	3/32
**Signal transduction: Activin A signaling regulation**	5.035x10^-4^	1.687x10^-2^	3/33
**Cell cycle: The metaphase checkpoint**	6.524x10^-4^	1.748x10^-2^	3/36
**Immune response: T regulatory cell-mediated modulation of effector T cell and NK cell functions**	2.023x10^-3^	4.176x10^-2^	3/53
**Apoptosis and survival: Endoplasmic reticulum stress response pathway**	2.250x10^-3^	4.176x10^-2^	3/55
**Transcription: Epigenetic regulation of gene expression**	2.493x10^-3^	4.176x10^-2^	3/57

Significantly enriched pathways in Cluster-1 include the endoplasmic reticulum stress pathway which is highly associated with RAG transcripts. Pathways with a FDR adjusted p-value less than 0.05 were considered significant. The ratio is indicative of the number of pathway objects (denominator) included in Cluster-1 (numerator).

While the remaining seven clusters did not fit with our *a priori* hypothesis, these data are available in S2 Table. Transcripts within these other seven clusters showed varying patterns in the degree of change and time course of gene expression. For example, Cluster-2 genes exhibited progressive downregulation from WK1 to WK2 that persisted through WK16. This cluster contained a number of transcripts associated with the DAergic phenotype. However, clusters 2–8 should be treated with caution due to a lack of *a priori* hypotheses regarding their patterns and the reality that consensus clustering can produce false clusters and inaccurate identification of the true *k* [36]. Due to the large number of genes being assessed and repeated sampling of the data there is a high probability that many of these same patterns could be found among a completely unrelated set of samples. Regardless, the differences in expression between lesioned and sham rats at each time point remain valid and should be further explored.

#### qPCR validation of genes from Cluster-1

**Fig 3:**
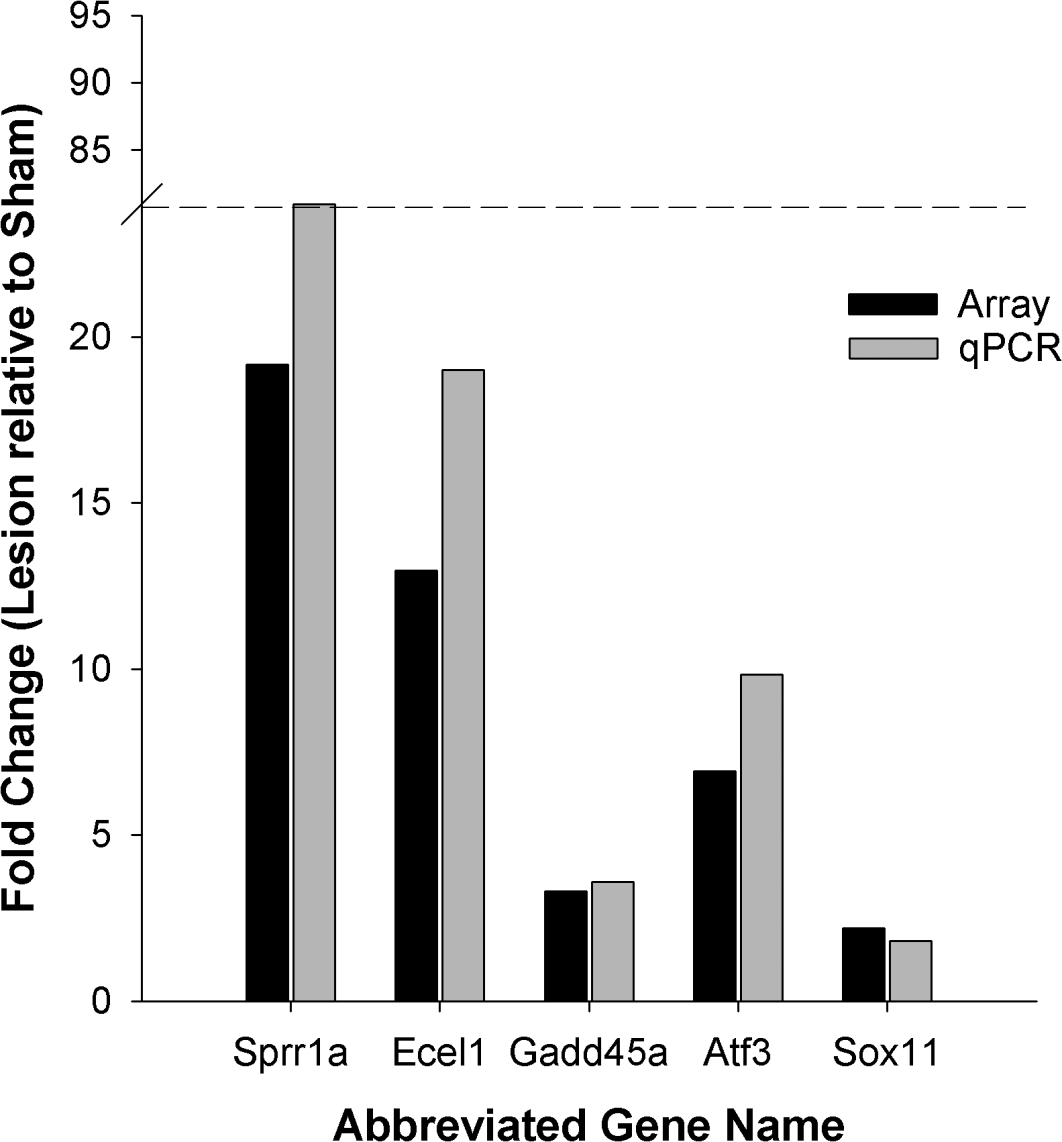
Exemplar Transcripts from Cluster-1 are validated by qPCR. The top 5 RAG transcripts from Cluster-1 were subjected to validation by qPCR. There was a high degree of agreement between the microarray and qPCR differential expression with the exception of Sprr1a. Differential expression of Sprr1a in the SN of 6-OHDA lesioned subjects was much greater when quantified by qPCR (80.3 fold) than originally observed by microarray (19.2 fold). This is likely due to saturation of the Sprr1a signal on the microarray chip, resulting in an underestimate of the differential upregulation.

The changes in expression levels for *Sprr1a*, *Ecel1*, *Gadd45*, *Atf3*, and *Sox11* were validated by qPCR in samples from the same 6-OHDA-WK1 and SHAM-WK1 subjects evaluated by microarray. These five RAG transcripts were chosen for validation due to the highly enriched presence of RAGs in Cluster-1 and were among the top six FDR adjusted p-values in Cluster-1. The qPCR confirmed a similar fold change in expression levels for, *Ecel1*, *Gadd45a*, *Atf3*, and *Sox11*. However *Sprr1a*, which demonstrated a 19 fold increase by microarray, exhibited an 80-fold increase by qPCR, suggesting that the microarray may have reached signal saturation (see Fig 3).

#### Demonstration that exemplar Cluster-1 transcripts were localized to DA neurons of the SNpc

**Fig 4:**
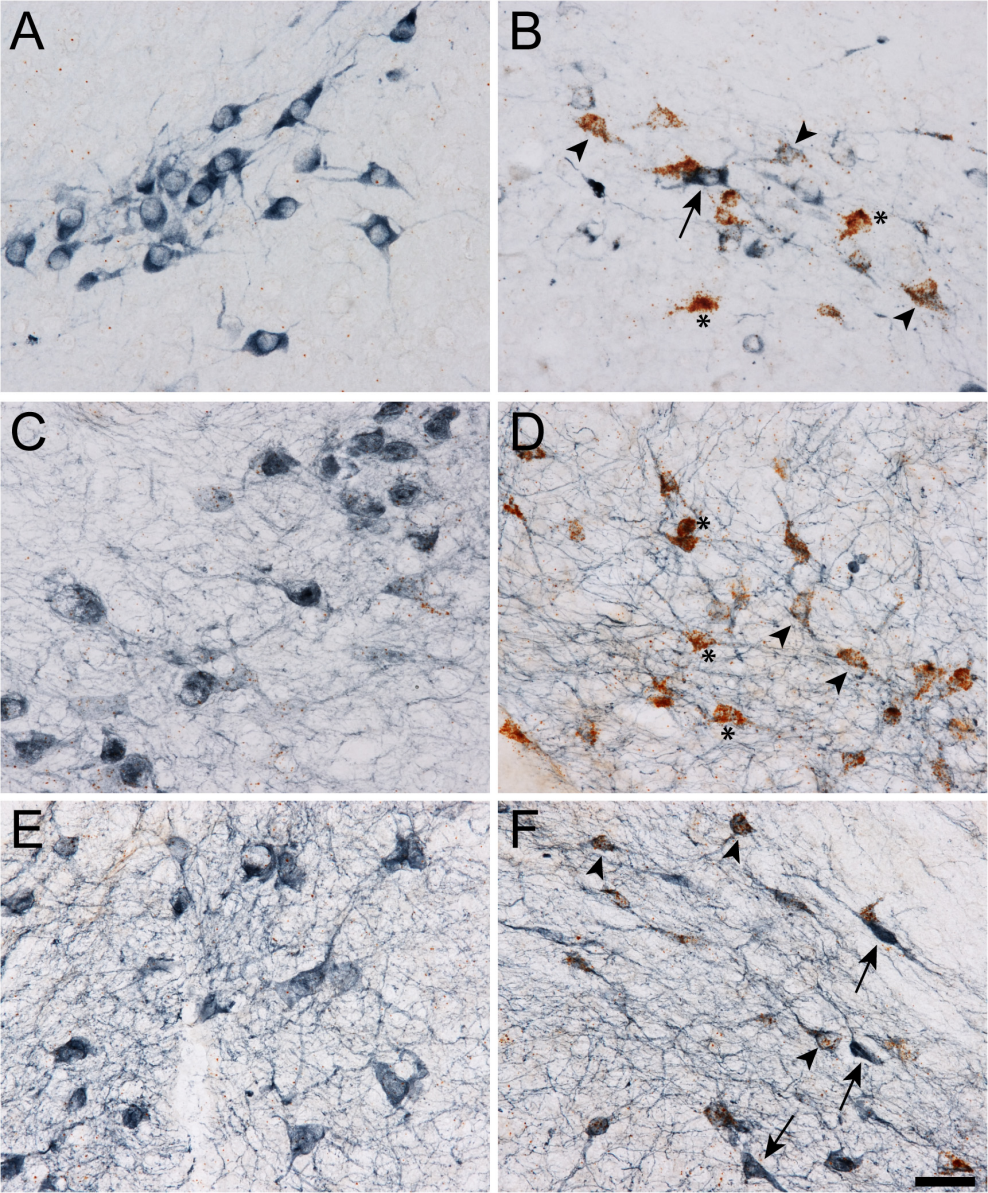
Upregulation of Sprr1a, Gadd45a and Sox11 early after intrastriatal 6-OHDA lesion occurs specifically in substantia nigra (SN) dopamine (DA) neurons. A-F). The mRNA for Sprr1a (A and B), Gadd45a (C and D) and Sox11 (E and F) was identified using RNAscope *in situ* hybridization (ISH; brown puncta) and DA neurons in the SN were identified with immunohistochemistry for tyrosine hydroxylase (blue staining, a DA neuron marker) in animals one week after an intrastriatal 6-OHDA lesion. The DA neurons on the intact side (i.e. contralateral to the 6-OHDA lesion) had little to no ISH signal due to the normally low basal expression of these RAGs (A, C, and E). In contrast, the DA neurons on the lesioned side had robust ISH signal (B, D and F). In the lesioned side, DA neurons appeared unhealthy and three cellular phenotypes were present, 1) little to no RAG ISH but strong TH indicating an apparently healthy neuron (arrow), 2) mild-moderate RAG ISH with moderate-weak TH staining indicating a DA neuron that is likely enroute to degenerating (arrowhead), and 3) robust ISH signal and little-no TH staining likely indicating DA neurons closer to degenerating (asterisk; DA neuron determination is based on neuroanatomical location within the SN and cell morphology). These data indicate that the upregulation of Sprr1a, Gadd45a and Sox11 identified by gene arrays and confirmed by qPCR occurred specifically in SN DA neurons. Scale bar = 50μm.

Next, we sought to confirm our hypothesis that the upregulation of genes attempting to confer axoprotection (i.e. RAGs) in Cluster-1 occurred specifically in DA neurons of the SN. We therefore chose three transcripts from Cluster-1 to pursue further using a combination of ISH to identify transcript changes in the RAGs and IHC to identify SN DA neurons. We observed a robust upregulation of *Sprr1a*, *Sox11* and *Gadd45a* transcripts (as indicated by RNAscope ISH) in TH+ SNpc neurons (Fig 4). These data suggest that, at least for the exemplar transcripts examined here, the progressive DA neuron loss from this intrastriatal 6-OHDA lesion is likely responsible for the progressive loss of signal over time in the transcripts of Cluster-1. These data also directly confirm that these RAGs are upregulated specifically in SN DA neurons in response to axonal degeneration by 6-OHDA.

### Network analysis of cCluster-1

**Fig 5:**
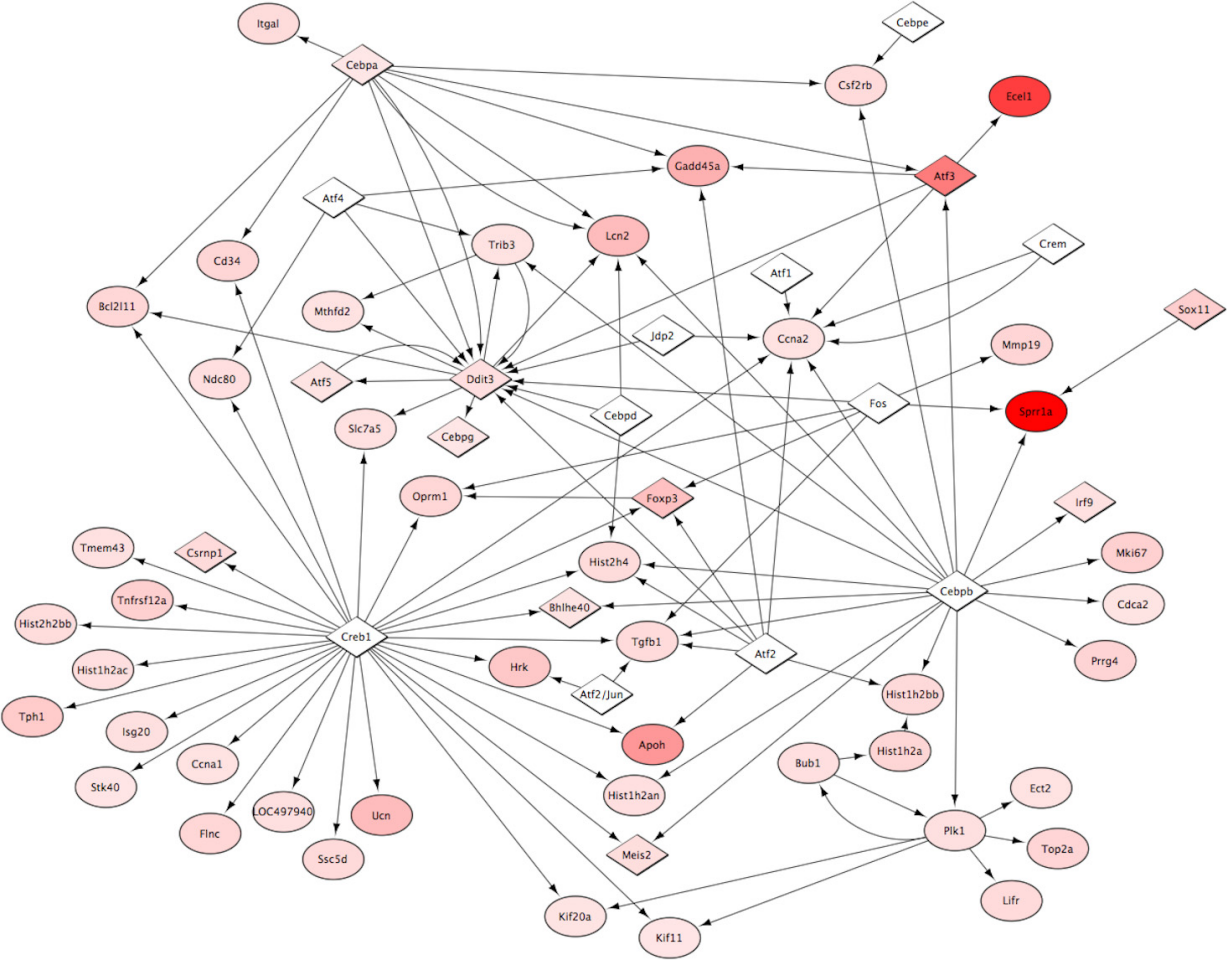
Pathway analysis demonstrates a high degree of interaction among the transcripts of Cluster-1. MetaCore Pathway analysis was conducted on the differential gene expression of Cluster-1 at 1 week post-surgery. The data were imported into Cytoscape for visualization. Diamonds represent transcription factors and ovals represent all other proteins. Arrows represent the direction of the interaction. The intensity of red coloring is indicative of the magnitude of upregulation. Values ranged from 1.23 (lightest pink) to 19.2 (darkest red). Of the 88 genes in Cluster-1, 62 had known interactions. The significant level of interaction among the transcripts along with the *a priori* hypothesis for the existence of Cluster-1 suggests this is a stable, biologically relevant cluster.

MetaCore network analysis demonstrated that transcripts from Cluster-1 were highly connected, with 62 of the transcripts showing evidence of interaction (Fig 5). This network included four significantly overconnected nodes (*Ddit3*; p = 4.14e-06, *Atf5*; p = 5.12e-05, *Atf3*; p = 0.001, Cebpg; p = 0.003, Mthdf3; p = 0.002) that are indicative of a response to stress [37,38].

## Discussion

In this study, we examined transcriptomic changes in the SN over 16 weeks after a unilateral, intrastriatal 6-OHDA lesion that results in progressive nigrostriatal DA axon degeneration followed by SN DA neuron loss [10]. Thus, this model replicates the early, profound nigrostriatal axon degeneration recently observed in post-mortem PD brains [11]. By using a novel k-means cluster analysis strategy, we generated clusters of transcripts defined by their covariation over time. This clustering suggests that groups of transcripts are responding to disconnection from the striatum in the same way or are interacting with each other in a coordinated fashion to respond to the 6-OHDA insult. Our *a priori* hypothesis was that genes expressed after induction of axonal degeneration would include potential axoprotective responses that are highly upregulated in the immediate/acute phases after injury but would then fall as overt loss of DA neurons occurs. In support of this hypothesis, we observed that transcripts with axon regenerative properties in the peripheral nervous system [12–15,24,30,39], including several members of the RAG family, are prominently upregulated early after insult in the SN followed by a rapid waning of expression (Cluster-1). The robust upregulation of a number of the RAGs identified by microarray were confirmed using qPCR, and in the case of *Sprr1a*, an increase of around 80 fold was observed.

The rapid induction and subsequent reduction in RAG and RAG-like expression over time indicates that these transcripts likely are (1) expressed in DA neurons of the SN and (2) their reduction over time is a result of the progressive degeneration of those DA neurons. Indeed, combing TH IHC with ISH for either *Sprr1a*, *Sox11* or *Gadd45a* confirmed that expression of at least these three RAG transcripts were substantially increased specifically in DA neurons of the SNpc (see Fig 4). While we cannot definitively conclude that the origin of all differentially expressed Cluster-1 transcripts in the SN come from DA neurons, our evidence supports the hypothesis that their rapid up-regulation is most likely the result of the DA axon damage that occurs in the initial stages of STR terminal damage. Future studies are needed to determine the cellular origin of the remaining member transcripts from Cluster-1. Additionally, we need to determine, prior to the one week time point, the temporal order of induction of Cluster-1 members in order to better understand the ordinal response of this gene cluster to axonal insult.

The specific RAG transcripts that we found upregulated in Cluster-1 include *Atf3*, *Sprr1a*, *Ecel1*, *Gadd45a*, *Gpnmb*, *Sox11*, *Mmp19*, *Srgap1*, *Rab15*,*Lifr*, *Trib3*, *Tgfb1*, and *Sema3c*. By definition, all of these transcripts are associated with a response to axonal damage and exhibit potential axoprotective or regenerative properties. Moreover, the majority of transcripts from Cluster-1 have significant interactions as illustrated in the network pathway analysis depicted in Fig 5, suggesting that other transcript members of the cluster may have RAG-like properties.

*Atf3* is among the more well-characterized RAGs in response to axonal damage. *Atf3* is upregulated in damaged dorsal root ganglion (DRG) neurons concurrent with neurite elongation *in vitro* [12]. Similarly, *Sprr1a* is relatively well-studied in peripheral neurons, and following DRG neuron axon damage it is significantly upregulated. The *Sprr1a* protein is associated with the axonal growth cone and is postulated to be integral to the growth and repair process of optic nerve and DRG neurons. Overexpression of *Sprr1a* enhances axonal recovery after injury, while reducing expression of *Sprr1a* impairs regenerative capabilities [14,15,24]. Sprr1a also known covaries with the transcription factor *Atf3* after axonal injury in cultured neurons [14]. Furthermore, neurite outgrowth is enhanced and *Sprr1a* expression is induced in cultured DRG neurons transfected with *Sox11* [15], suggesting that *Sox11* is a transcription factor for *Sprr1a*. In this regard, the present TH/*Sox11* and TH/*Sprr1a* immunostaining analysis demonstrates that both of these RAGs are highly upregulated in damaged DA neurons (Fig 4). This novel observation indicates that the response of SN DA neurons to axonal injury is under the control of many of the same factors observed in DRG neurons. Functionally, *Sox11* may regulate expression of cytoskeletal proteins (e.g. βIII-tubulin, MAP2 and doublecortin), while *Sprr1a* may directly interact with structural cytoskeleton proteins critical (e.g. actin; for a review see [40]).

Another member of the RAG family induced by axonal injury in nigrostriatal DA neurons is *Gadd45a*. Spinal cord injury induces *Gadd45a* expression [41,42] in both sensory and motor neurons of the spinal cord. Neuronal damage activates the JNK-p53-GADD45α cascade and *Gadd45a* inhibition may be a potential neuroprotective strategy [25], whereas another study suggests that *Gadd45a* may be neuroprotective as its overexpression can prevent neurodegeneration [13]. The underlying cause of these discrepant findings is unclear and whether modulation of *Gadd45a* expression will affect DA neuron degeneration awaits future studies.

Intriguingly, parkin overexpression induces *Gadd45a* expression *in vitro* suggesting a link between some forms of parkinsonism and this transcript [43]. With respect to other RAGs in Cluster-1, *Ecel1* (aka, *DINE*) is upregulated in response to DRG injury [22] and injury to retinal ganglion cells induces *Ecel1*, *Atf3*, *Gadd45a* and *Sprr1a* [30]. Once again, these data demonstrate the highly interrelated response of RAGs to injury among a wide variety of neuronal cell types.

Other transcripts that behaved similar to established RAGs are less well characterized yet may play a significant role in the response to axonal damage in DA neurons. For instance, *Mmp19* expression was only recently found in neurons and is speculated to promote axonal growth and remodeling [31]. *Lifr*, the alpha receptor for leukemia inhibitory factor, also has axon regenerative properties in retinal ganglion cells [39]. *Srgap1* interacts with axonal guidance molecules during development [39] and plays a potentially important role in neurite outgrowth [32]. Likewise, the small GTPase *Rab15* is implicated in neuronal differentiation [39]. *Tgfb1*, has anti-apoptotic, anti-inflammatory and pro-myelin maintenance and production properties [35,44,45]. *Gpnmb* may show similar functions as it was recently identified as a potential ALS-related factor in the spinal cords of SOD1^G93A^ mice [26]. Another RAG-like interaction of note is between Trib3 and Atf4. Trib3 is a pseudokinase that is upregulated in response to neuronal death and increases the activity of Atf4 [33,34]. Atf4, in turn, is implicated in the axodegenerative process [46], suggesting that in our model Trib3 upregulation might signal antagonistic axoprotective responses such as RAG induction. Finally, Sema3c is an axonal guidance molecule that is part of the semaphorin family of genes which are involved in axonal navigation and pathfinding in development and neuronal injury [23,27–29,47]. The remainder of Cluster-1 warrants thorough examination for potential roles in the coordinated response of the SNc to DA axon damage, due to their similar RAG-like regulation profile.

Exactly what signals cause the upregulation of RAG and RAG-like transcripts requires further investigation. Interestingly, several transcription factors that are implicated in the ER stress response show significant pathway interaction as indicated by MetaCore analysis (*Ddit3*, *Atf5*, *Atf3*, *Cebpg*)[37,38]. An important gene connected to these transcription factors is *Bcl2l11*, a known important mediator of apoptosis [48]. Understanding which of these stress-induced transcripts are signals of cellular trauma, and which are transducers of axoprotective responses will be critical in clarifying their complex, coordinated interrelationship. This understanding could lead to new targets for the development of axoprotective therapeutics.

Notably, the SN RAG transcripts up-regulated in response to striatal 6-OHDA administration were not previously described in DA neurons. For many of these transcripts, this is the first time they were observed to be upregulated in response to CNS injury [12,13,24,30,39,49–53]. This striking observation suggests a conservation of genetic axoprotective programs within the nervous system. Furthermore, these data underscore the importance of these gene pathways as critical mediators of axonal maintenance and regrowth after injury in nigrostriatal DAergic neurons.

Finally, it should be restated here that while there is a wealth of data demonstrating differential gene expression across genes from all eight clusters, one must be cautious in drawing conclusions about the association of genes within any particular cluster without an *a priori* hypotheses due to a high probability of false positive associations. As a result, new hypotheses could be proposed regarding the longitudinal response to intrastriatal lesioning as long as the appropriate caveats are acknowledged.

Recent discoveries regarding the early and robust nature of axonal degeneration in the DAergic nigrostriatal pathway that precedes overt DA neuron loss in the SN highlights the importance of targeting axonal degeneration in treating PD. In light of this recent observation [11], the data presented here provide potential new avenues to pursue toward target development to prevent or reverse nigrostriatal axon degeneration. The fact that many of the RAG changes are attributable to the DA neurons themselves very early after 6-OHDA insult suggest that transcript specific responses of DA neurons are attempting to resist and repair axonal degeneration. Moving forward, the critical next step is to perform studies aimed at determining whether modulation of the identified DA neuron-derived RAGs and RAG-like transcripts can provide axoprotection and resultant neuroprotection in SN DA neurons. These novel transcript responses identified in DA neurons may result in new strategies for preventing the early axonal loss observed in PD patients [11].

## Supporting Information

S1 Table Fold changes at each time point, F-statistics and p values for the transcripts within Cluster-1. (TXT)

S2 Table Cluster assignment (1–8) and t-statistic at each time point for differentially expressed transcripts from the microarray analysis. (TXT)
